# Is d-aspartate produced by glutamic-oxaloacetic transaminase-1 like 1 (Got1l1): a putative aspartate racemase?

**DOI:** 10.1007/s00726-014-1847-3

**Published:** 2014-10-07

**Authors:** Ayumi Tanaka-Hayashi, Shuuhei Hayashi, Ran Inoue, Tomokazu Ito, Kohtarou Konno, Tomoyuki Yoshida, Masahiko Watanabe, Tohru Yoshimura, Hisashi Mori

**Affiliations:** 1 Department of Molecular Neuroscience, Graduate School of Medicine and Pharmaceutical Sciences, University of Toyama, Toyama, 930-0194 Japan; 2 Department of Applied Molecular Biosciences, Graduate School of Bioagricultural Sciences, Nagoya University, Chikusa-Ku, Nagoya, Aichi 464-8601 Japan; 3 Department of Anatomy, Graduate School of Medicine, Hokkaido University, Sapporo, 060-8638 Japan

**Keywords:** Glutamic-oxaloacetic transaminase-1 like 1, d-Aspartate, Knockout mice, Testis, Hippocampus, Recombinant protein expression

## Abstract

d-Aspartate is an endogenous free amino acid in the brain, endocrine tissues, and exocrine tissues in mammals, and it plays several physiological roles. In the testis, d-aspartate is detected in elongate spermatids, Leydig cells, and Sertoli cells, and implicated in the synthesis and release of testosterone. In the hippocampus, d-aspartate strongly enhances *N*-methyl-d-aspartate receptor-dependent long-term potentiation and is involved in learning and memory. The existence of aspartate racemase, a candidate enzyme for d-aspartate production, has been suggested. Recently, mouse glutamic-oxaloacetic transaminase 1-like 1 (Got1l1) has been reported to synthesize substantially d-aspartate from l-aspartate and to be involved in adult neurogenesis. In this study, we investigated the function of Got1l1 in vivo by generating and analyzing *Got1l1* knockout (KO) mice. We also examined the enzymatic activity of recombinant Got1l1 in vitro. We found that *Got1l1* mRNA is highly expressed in the testis, but it is not detected in the brain and submandibular gland, where d-aspartate is abundant. The d-aspartate contents of wild-type and *Got1l1* KO mice were not significantly different in the testis and hippocampus. The recombinant Got1l1 expressed in mammalian cells showed l-aspartate aminotransferase activity, but lacked aspartate racemase activity. These findings suggest that Got1l1 is not the major aspartate racemase and there might be an as yet unknown d-aspartate-synthesizing enzyme.

## Introduction

Some free d-amino acids exist at high concentrations in several tissues in mammals. d-Aspartate is present in some tissues such as those in the hippocampus, pineal body, and pituitary in the brain, the submandibular gland (SMG), and the testis (Furuchi and Homma 2005; Hashimoto and Oka 1997; Masuda et al. 2003). In the hippocampus, d-aspartate can strongly enhance *N*-methyl-d-aspartate receptor (NMDAR)-dependent long-term potentiation (LTP) and rescue age-related deficiency in synaptic plasticity (Errico et al. 2011). In addition, a high level of d-aspartate is related to learning and memory (Topo et al. 2010). d-Aspartate is also involved in the synthesis and release of the luteinizing hormone in the pituitary and melatonin release in the pineal body (D’Aniello et al. 2000; Ishio et al. 1998; Takigawa et al. 1998). In the testis, d-aspartate is detected in elongated spermatids, Leydig cells, and Sertoli cells, involved in the synthesis and release of testosterone (D’Aniello et al. 1996; Sakai et al. 1998).

Although several studies have shown the functions of d-aspartate, the enzyme producing d-aspartate was not identified in mammals. Wolosker et al. (2000) have revealed that d-aspartate is synthesized from l-aspartate in the primary neuronal cultures derived from rat embryos, suggesting the existence of mammalian aspartate racemase in neurons. Kim et al. (Kim et al. 2010) reported that glutamic-oxaloacetic transaminase 1-like 1 (Got1l1) is the mouse aspartate racemase, and Got1l1 is localized in paraventricular nuclei (PVN), supraoptic nuclei (SON), and hippocampal neurons of the adult mouse brain. A recombinant Got1l1 protein is a pyridoxal 5′-phosphate (PLP)-dependent enzyme and synthesizes substantial d-aspartate from l-aspartate and only one-fifth as much l-glutamate, with very little d-glutamate in vitro. Depletion of *Got1l1* by short-hairpin RNA in newborn neurons of the adult hippocampus induces defects in dendritic development and survival of newborn neurons. However, the function and contribution of *Got1l1* as an aspartate racemase has not been clarified in vivo.

In the present study, we generated *Got1l1* knockout (KO) mice. We found that *Got1l1* mRNA was highly expressed in the testis in wild-type (WT) mice, but was not detected in *Got1l1* KO mice. The d-aspartate contents of WT and *Got1l1* KO mice were not significantly different in the testis and hippocampus. We also examined the enzymatic activity of the recombinant *Got1l1* prepared from *Escherichia coli* (*E. coli*) and HEK293T cells. Got1l1 catalyzed no aspartate racemization, but catalyzed the transamination between l-aspartate and α-ketoglutarate and produced small amount of l-glutamate under the conditions examined.

## Materials and methods

### Generation of *Got1l1* KO mice

Animal care and experimental protocols were approved by the Animal Experiment Committee of the University of Toyama (Authorization No. 2012 med-41) and were carried out in accordance with the Guidelines for the Care and Use of Laboratory Animals of the University of Toyama.

A bacterial artificial chromosome (BAC) clone (B6Ng01-118F09) containing mouse *Got1l1* was provided by RIKEN BRC through the National Bio-Resource Project of the Ministry of Education, Culture, Sports, Science and Technology (MEXT), Japan. The nucleotide sequence of the mouse genome was obtained from the National Center for Biotechnology Information (NCBI) (Mouse G+T, Annotation Release.103).

For the construction of the *Got1l1*-targeting vector, the 5′ homology arm of 5.2 kb (−3,648 to +1,533; the nucleotide residues of the mouse BAC clone are numbered in the 5′–3′ direction, beginning with the A of ATG, the initiation site of translation in *Got1l1*, which refers to position +1, and the preceding residues are indicated by negative numbers) and 3′ homology arm of 6.9 kb (+1,800 to +8,730) from the BAC clone were subcloned into the pDONR P4-P1R and pDONER P2R-P3 vectors (Invitrogen, Carlsbad, CA), respectively, using a counter-selection BAC modification kit (Gene Bridges, Dresden, Germany). The 266-bp DNA fragment (+1,534 to +1,799) containing *Got1l1* exon 2 was amplified by PCR and subcloned between two *loxP* sites of a modified pDONR221 vector containing a phosphoglycerate kinase (pgk) promoter-driven neomycin cassette (pgk-neo) flanked by two FRT sites. These three plasmids were directionally subcloned into pDEST R4-R3 containing the diphtheria toxin gene (MC1-DTA) using LR clonase of a MultiSite Gateway Three-Fragment Vector Construction kit (Invitrogen, Carlsbad, CA) to yield the targeting vector. The targeting vector linearized with *Not*I was electroporated into the embryonic stem (ES) cell line RENKA (Fukaya et al. 2006) derived from the C57BL/6N strain as previously described (Mishina and Sakimura 2007; Miya et al. 2008). After the selection with G418, the recombinant ES clone (No. 51) was identified by Southern blot analysis using the 5′ outer probe (−4,407 to −3,807) and 3′ outer probe (+8,821 to +9,416) on *Nsi*I-digested genomic DNA, and the Neo probe (Miya et al. 2008) on *Kpn*I-digested genome DNA. To delete exon 2 of *Got1l1* flanked by *loxP* sites, a circular pCre-Pac plasmid (10 μg) expressing Cre recombinase (Taniguchi et al. 1998) was electroporated into the obtained recombinant ES clone.

The obtained ES clone was injected into eight-cell stage embryos from ICR mice. The embryos were cultured to the blastocyst stage and transferred to the uterus of pseudopregnant ICR mice. The resulting male chimeric mice were crossed with female C57BL/6 N mice to establish the mutant mouse line.

### Northern blot analysis

Northern blot analysis was performed as previously reported (Miya et al. 2008). In brief, total RNA samples were prepared, using TRIsol Reagent (Invitrogen, Carlsbad, CA), from the brain, testis, SMG, and liver of WT and *Got1l1* KO mice, separated by agarose gel electrophoresis, and blotted on membranes (Hybond N^+^; GE Healthcare, Buckinghamshire, UK). Blotted membranes were hybridized with a ^32^P-labeled *Got1l1* cDNA fragment corresponding to exons 1 and 2 or a β-actin cDNA fragment corresponding to the protein-coding region.

### Measurement of amino acid contents in the testis and hippocampus

To decrease the effect of amino acids derived from food on the amino acid content in the testis and brain, each male mouse was fasted for 24 h before sampling. The mice were deeply anesthetized with pentobarbital (65 mg/kg body weight, intra-peritoneal), perfused transcardially with PBS (pH 7.4). The testes and hippocampi were removed, weighed, and frozen in liquid N_2_. These organs were homogenized in 10 vol. (ml/g) of 0.2 M trichloroacetic acid (TCA) and the debris was removed by centrifugation. TCA was removed by extraction three times, with water-saturated diethyl ether. The amino acid contents were determined by HPLC as previously described (Ito et al. 2008) with a slight modification. When necessary, d-threo-3-hydroxy aspartate (d-THA) was used as an internal standard. Mobile phase A consisted of 9 % acetonitrile in 0.1 M acetate buffer (pH 6.0), and mobile phase B was 50 % acetonitrile in 0.1 M acetate buffer (pH 6.0). A linear gradient of the mobile phase B was developed from 0 to 7.5 % between 0 and 10 min, 7.5–17.5 % between 10 and 35 min, and 17.5–30 % between 35 and 60 min.

### Fluorescence in situ hybridization

The cDNA clones of mouse *Got1l1* and human *Got1l1* (*hGot1l1*) were purchased from DNAFORM (clone ID Nos.: 6533656 for *Got1l1* and H013075A20 for *hGot1l1*). To produce the RNA probe, the cDNA fragment of the mouse *Got1l1*-coding region (No. 62-1276; NCBI Reference Sequence NM_029674.1), was amplified from the above-mentioned cDNA clone and subcloned into the pBluescript II plasmid vector (Invitrogen, Carlsbad, CA, USA). cRNA probes were prepared as described previously (Yamazaki et al. 2010).

Section preparation and fluorescence in situ hybridization were performed as described previously (Kudo et al. 2012). Fluorescence was detected using a Cy3-TSA plus amplification kit (PerkinElmer. Inc. MA, USA). Finally, sections were counterstained with the fluorescent nuclear stain TOTO3 (Molecular Probes, Eugene, USA).

### Construction and expression of GST-fused Got1l1

The mouse and human *Got1l1* cDNAs were amplified by PCR and cloned into the pGEX-4T vector (GE Healthcare). The resultant plasmids named pGEX-*Got1l1* and pGEX-*hGot1l1* expressed Got1l1 with N-terminally glutathione *S*-transferase (GST)-tagged fusion proteins. The *E. coli* BL21 cells (Novagen, Madison, WI, USA) transformed with each plasmid were cultivated in an LB medium containing 100 μg/ml ampicillin at 22 °C. The protein was expressed by adding 0.1 mM isopropyl β-d-thiogalactopyranoside (IPTG) at the mid-log phase. The GST fusion protein was purified by affinity chromatography with glutathione Sepharose 4B resin (GE Healthcare) in accordance with the manufacturer’s instruction.

The d-glutamate auxotrophy assay was performed as described previously (Doublet et al. 1992). We used *E. coli* WM335 cells harboring the empty vector (pGEX-4T), pGEX-*Got1l1*, pGEX-*hGot1l1*, or pICT113 expressing d-amino acid aminotransferase. These *E. coli.* WM335 cell strains were cultivated with or without 0.1 % d-glutamate on LB agarose plates containing ampicillin and 0.1 mM IPTG. The plates were incubated at 22 °C for 3 days and photographed.

### Enzymatic activity of recombinant Got1l1

HEK293T cells transfected with an expression vector for C-terminally histidine (His)-tagged Got1l1 (pEB-Got1l1-His) and an empty vector (pEB6) were collected from two Petri dishes (10 cm diameter) each for the preparation of recombinant Got1l1 and control cell extract, respectively. The cells were sonicated in 400 μl of lysis buffer consisting of 50 mM Tris–HCl (pH 7.4), 150 mM NaCl, 1 % Triton X-100, and protease inhibitors cocktail lacking EDTA (nacalai tesque, Kyoto, Japan), and centrifuged at 20,000×*g* for 30 min. The supernatant was incubated with Ni-Sepharose 6 fast flow resin (GE Healthcare) for 4 h at 4 °C. After the resin was washed five times with wash buffer (PBS containing 10 mM imidazole and 0.5 % Triton X-100), proteins were eluted with 500 μl of elution buffer (PBS containing 1 M imidazole). The eluates were immediately passed through a PD MiniTrap G-25 column (GE Healthcare) equilibrated with PBS. Then, a 100-μl aliquot of the protein solution was mixed with the same volume of the reaction mixture consisting of 50 mM Tris–HCl (pH 7.5), 40 μM PLP, 10 mM l-aspartate, and 10 mM α-ketoglutarate. After 4 h incubation at 37 °C, the reaction was terminated by adding 200 μl of 0.2 M TCA. The formation of d-aspartate, d-glutamate, and l-glutamate was examined by HPLC as described above.

## Results

### Expression of *Got1l1* mRNA in several tissues

**Fig. 1:**
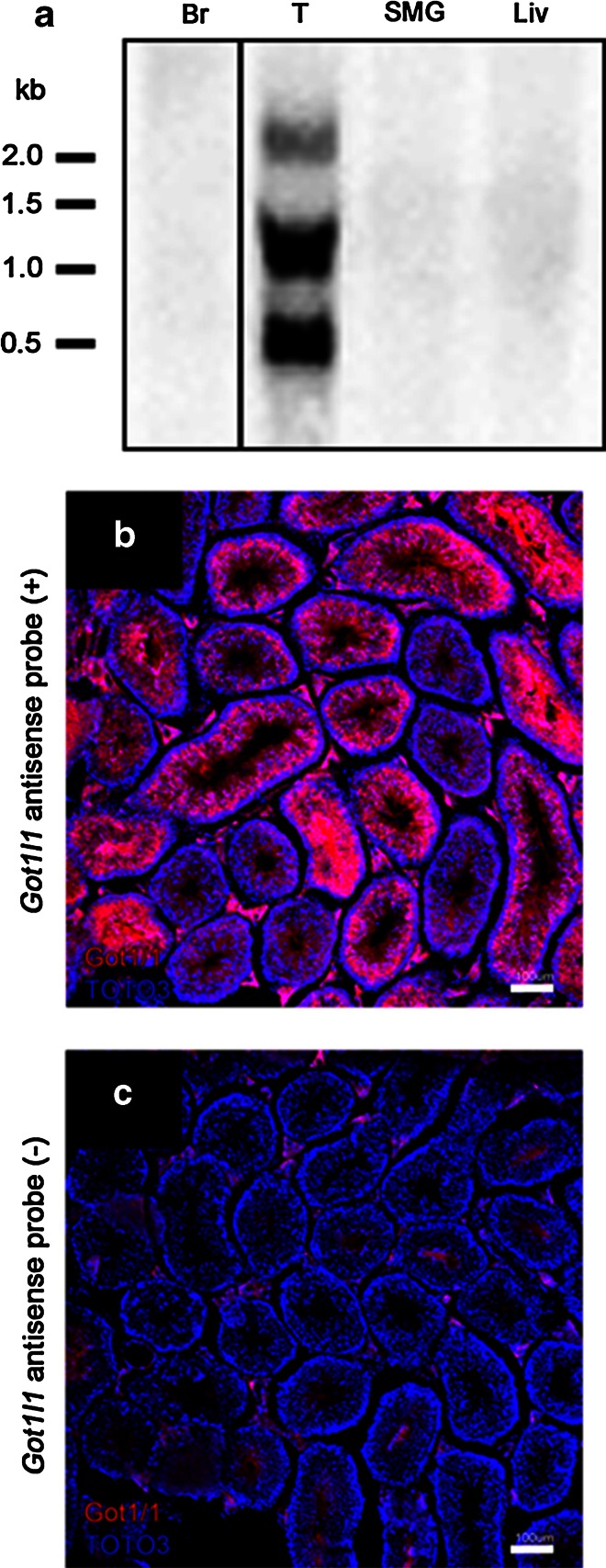
mRNA expression of *Got1l1* in several tissues. **a** Northern blot analysis of total RNA (10 μg) extracted from the brain (Br), testis (T), submandibular gland (SMG), and liver (Liv) of WT mouse using the *Got1l1* exon 1–2 probe. The positions of RNA size markers are indicated on the *left side*. Br *lane* is from different part of the same blot. **b**, **c** Images of fluorescence in situ hybridization of the testis with (**a**) or without (**b**) *Got1l1* antisense probe. Red signals of *Got1l1* mRNA are detected in spermatocytes and sperms. Some red signals observed in Leydig cells and the lumen of seminiferous tubules without the probe (**c**) are nonspecific signals. The sections were counterstained with TOTO3 (*blue*) for nuclear visualization. The *bars* indicate 100 µm

To examine the expression of *Got1l1* mRNA, we performed Northern blot analysis using total RNA from tissues of the brain, testis, and SMG, because these tissues were reported to contain abundant d-aspartate in mice and rats (Errico et al. 2012; Masuda et al. 2003; Sakai et al. 1998). We detected the main band of about 1.2 kb, in addition to the bands of 0.7 and 2.2 kb in total RNA from the testis (Fig. 1a). These bands corresponding to *Got1l1* mRNA were not detected in total RNA from the brain, SMG, and liver.

To examine the distribution of *Got1l1* mRNA, we performed in situ hybridization analysis using fluorescence-labeled *Got1l1* antisense RNA probe. Specific *Got1l1* mRNA signals were detected in primary spermatocytes and sperms in the testis (Fig. 1b, c). We were not able to detect specific *Got1l1* mRNA signals in the adult hippocampus and other brain regions (data not shown).

### Generation of *Got1l1* KO mice

**Fig. 2:**
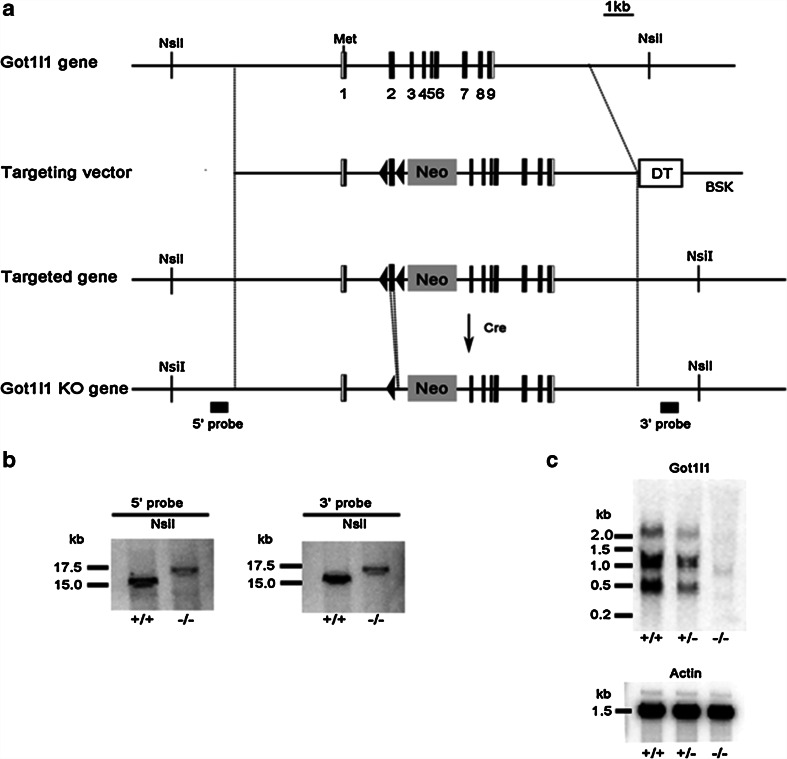
Generation of *Got1l1* knockout mouse. **a** Schematic representation of *Got1l1* gene, targeting vector, targeted gene, and *Got1l1* KO gene. The coding and noncoding regions of *Got1l1* exons are indicated by *closed* and *open boxes*, respectively. The inserted pgk-neo gene (Neo) is shown. Met is the initiation site of translation in *Got1l1*. The restriction enzyme sites (*Nsi*I) and location of probes used (5′ and 3′ probe) are indicated. *Black triangles* indicate *loxP* sites. **b** Southern blot analysis of genomic DNA (8 μg) from WT (+/+) and Got1l1 KO (−/−) mice. *Nsi*I-digested genomic DNA was hybridized with the 5′ outer probe (*left*) and 3′ outer probe (*right*). The positions of DNA size markers are indicated on the *left side*. **c** Northern blot analysis of total RNA (10 μg for *Got1l1* probe, 1 μg for *Actin* probe) extracted from the testes of WT (+/+), heterozygous (+/−), and Got1l1 KO (−/−) mice. The hybridizations were carried out using the *Got1l1* exon 1–2 probe (*upper*) and *actin* probe (*lower*). The positions of RNA size markers are indicated on the *left side*

To investigate the function of Got1l1 in vivo, we generated *Got1l1* KO mice (Fig. 2a). We constructed a targeting vector in which exon 2 of *Got1l1* was flanked by two loxP sequences followed by a pgk-neo selection marker. After the selection using G418, the correctly targeted embryonic stem (ES) cell clone (No. 51) was identified by Southern blot analysis using 5′- and 3′-outer probes (data not shown). To delete the region between the loxP sequences and to generate a frame-shift mutation of a *Got1l1* allele (*Got1l1* KO), a circular pCre-PAC plasmid (Taniguchi et al. 1998) was transiently introduced into the ES clone. Chimeric mice derived from this ES clone were mated with C57BL/6 N mice to establish the mutant mouse line. After crossing between heterozygous mutant mice, we obtained *Got1l1* KO mice identified by Southern blotting (Fig. 2b). The expression of *Got1l1* mRNA in the testis was analyzed by Northern blotting. All the bands detected for WT mice showed a decreased intensity for heterozygous mice and were not detectable for the *Got1l1* KO mice (Fig. 2c). The *Got1l1* KO mice were viable and fertile.

### Comparison of amino acid contents of testis and hippocampus between WT and *Got1l1* KO mice

**Fig. 3:**
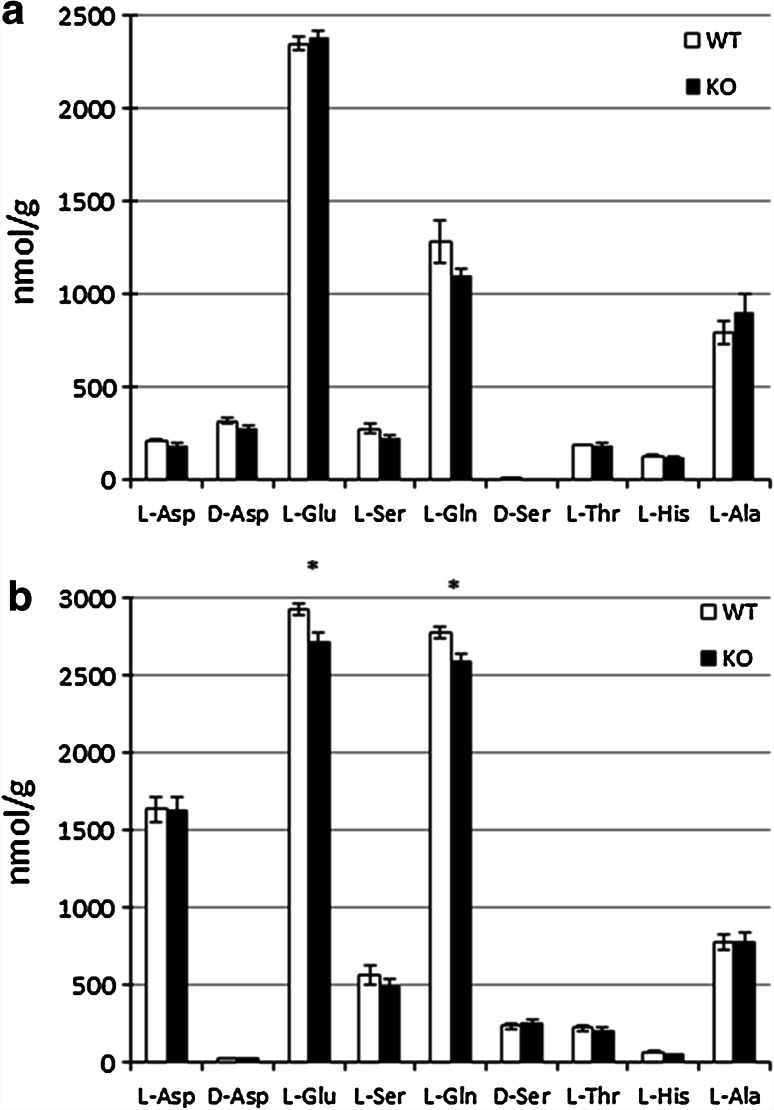
Contents of amino acids in WT and *Got1l1* KO mice. *Bar graphs* show contents (nmol/g) of amino acids in testes (**a**) (*n* = 4 each) and hippocampi (**b**) (*n* = 8 each) of WT and *Got1l1* KO mice measured by HPLC *p < 0.05

We examined the amino acid contents of the testis in adult WT (*n* = 4) and *Got1l1* KO (*n* = 4) mice by HPLC. There were no significant differences in d-aspartate content between the two genotypes (Student’s *t* test, *p* = 0.21). Furthermore, the contents of other l- and d-amino acids examined were also comparable between the two genotypes (Fig. 3a).

Next, we investigated the amino acid contents of the hippocampus from adult WT (*n* = 8) and *Got1l1* KO (*n* = 8) mice. Although we did not detect a significant difference in the content of d-aspartate between the two genotypes, we found that the contents of l-glutamate and l-glutamine slightly but significantly decreased in the *Got1l1* KO mice (*p* < 0.05) (Fig. 3b).

### Aspartate racemase activity of recombinant Got1l1 protein

The above-mentioned findings suggest that Got1l1 is not a major d-aspartate-producing enzyme. We, therefore, attempted to confirm the enzymatic activity of Got1l1. In accordance with a previous report (Kim et al. 2010), we constructed several mouse Got1l1 overexpression vectors using *E. coli* pET-vector systems, pET15b, and pET22b. Got1l1 was expressed with an N-terminal His tag, with a C-terminal His tag, and without His tag. Unlike in the previous report (Kim et al. 2010), we were unable to obtain Got1l1 as a soluble protein. Various attempts to obtain soluble Got1l1, such as alteration of host cells, coexpression with chaperons, cultivation at low temperatures, or slow induction using a low concentration of an inducer, did not increase the solubility of Got1l1. Similar results were obtained for human Got1l1 (hGot1l1).

**Fig. 4:**
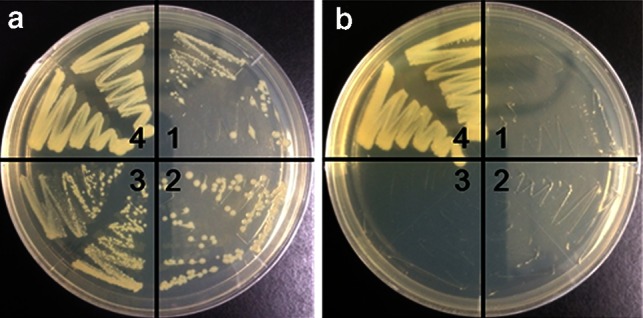
d-Glutamate-producing activity of Got1l1 in *E. coli* WM335 cells. *E. coli* WM335 cells harboring the empty vector (pGEX-4T) (*1*), pGEX-*Got1l1* (*2*), pGEX-*hGot1l1* (*3*), or pICT113 expressing d-amino acid aminotransferase (*4*) were cultivated with (**a**) or without (**b**) 0.1 % d-glutamate on an LB plate containing ampicillin and 0.1 mM IPTG at 22 °C for 3 days

We, therefore, changed the expression system from a pET-vector system to a pGEX-vector system, and Got1l1 was expressed as an N-terminally glutathione *S*-transferase (GST)-tagged fusion protein in *E. coli* cells. A small amount of GST-Got1l1 was obtained in a soluble fraction of the *E. coli* cell lysate when it was expressed at a low temperature (22 °C). We incubated the purified GST-Got1l1 with l-aspartate, but no d-aspartate was formed under the conditions examined (data not shown). Kim et al. (Kim et al. 2010) reported that Got1l1 produces d-glutamate in the presence of l-aspartate and α-ketoglutarate. d-Glutamate production was thus examined using GST-Got1l1 in the presence of l-aspartate and α-ketoglutarate, but no detectable amount of d-glutamate was produced (data not shown). During the reaction, we detected the formation of a small amount of l-glutamate. However, we could not rule out the possibility that l-glutamate was produced by a contaminating transaminase from the *E. coli* host cells. The enzymatic activity of Got1l1 was also examined using GST-fused hGot1l1 (GST-hGot1l1). However, neither aspartate racemase activity nor d-glutamate-producing activity was observed for GST-hGot1l1 in vitro.

d-Glutamate production using pGEX-*Gotl1l* and pGEX-*hGot1l1* was also examined using *E. coli* WM335 showing d-glutamate auxotrophy (Doublet et al. 1992). The d-glutamate auxotrophy of *E. coli* WM335 can be compensated for by expressing of d-amino acid aminotransferase or other enzymes catalyzing the d-glutamate formation although inefficiently (Liu et al. 1998). As shown in Fig. 4, the expression of GST-Got1l1 (and also GST-hGot1l1) did not complement the d-glutamate auxotrophy of *E. coli* WM335. These findings suggest that GST-Got1l1 catalyzes little or no d-glutamate formation in the *E. coli* WM335 cells under the conditions used in this study.

**Fig. 5:**
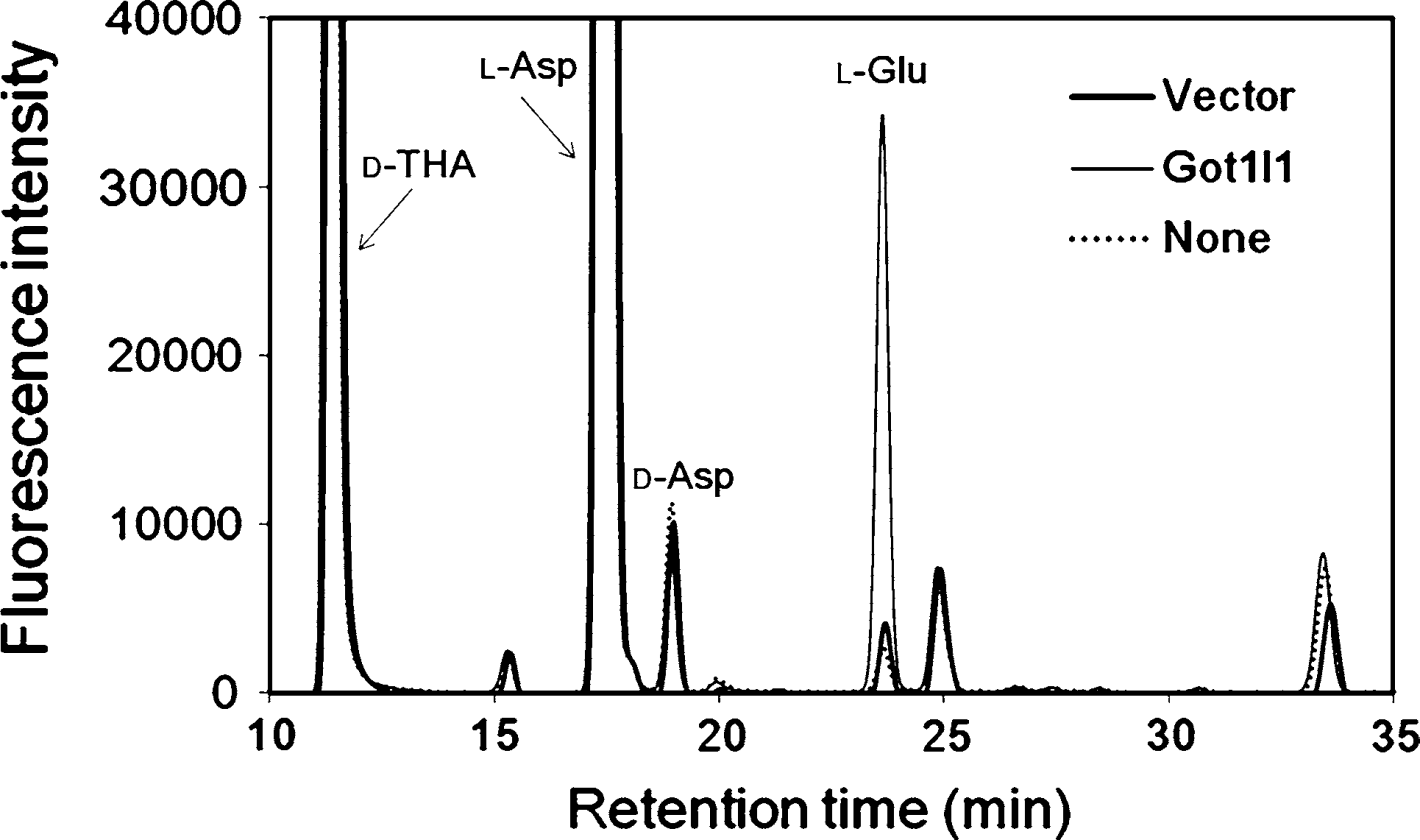
Enzymatic activity of recombinant Got1l1. Enzymatic activity of Got1l1 prepared from HEK293T cells was examined. Got1l1 purified from the HEK293T cells harboring an expression vector for Got1l1 (Got1l1), the control cell extract from the cells containing an empty vector (Vector), or PBS (None) was incubated with l-aspartate and α-ketoglutarate for 4 h at 37 °C. The final concentration of Got1l1 in the reaction mixture was approximately 0.5 μg/ml. The reaction was stopped by adding of TCA and the amino acid contents were determined by HPLC. d-THA was added before HPLC as an internal standard

We also confirmed the activity of the C-terminally His-tagged Got1l1 expressed in HEK293T cells. The recombinant Got1l1 could be obtained as a soluble protein from HEK293T cells and was batch-purified using a Ni-affinity resin. The presence of Got1l1 was confirmed by western blot analysis using an anti-His-tag antibody. The extract was reacted with l-aspartate, and the resultant reaction mixture was analyzed by HPLC. However, we obtained no detectable amount of d-aspartate. When l-aspartate and α-ketoglutarate were reacted with the Got1l1 purified from HEK293T cells, neither d-aspartate nor d-glutamate was generated, but a detectable amount of l-glutamate was obtained (Fig. 5). When the control cell extract was used instead of Got1l1, l-glutamate was not obtained. These findings strongly suggest that the recombinant Got1l1 has neither aspartate racemase activity nor d-glutamate-producing activity, but has l-aspartate aminotransferase activity in vitro.

## Discussion

In a previous study, Kim et al. (2010) demonstrated that cloned Got1l1 synthesizes substantial d-aspartate, and only one-fifth as much l-glutamate, with very little d-glutamate in vitro. Their results suggest that Got1l1 has aspartate racemase activity, and low aminotransferase activity. However, in this study, we failed to detect Got1l1-catalyzed aspartate racemase activity. Unlike in the previous study, the recombinant Got1l1 was not obtained in a soluble fraction of *E. coli* when the *E. coli* pET-vector system was used. GST-fused Got1l1 was obtained as a soluble protein, but it lacked aspartate racemase activity both in vitro and *in E.coli*. Essentially, the same results were obtained with human Got1l1. The recombinant Got1l1 prepared from HEK293T cells were found to have an l-aspartate aminotransferase activity, but have no aspartate racemase activity in vitro. Although we used relative small amounts of the recombinant Got1l1 protein as compared with previous report (Kim et al. 2010), we detected significant l-aspartate aminotransferase activity. However, we could not detect any d-aspartate racemase activity. Furthermore, we found that there was no significant difference in d-aspartate contents in the testis and hippocampus from WT and *Got1l1* KO mice.

We detected a high level of *Got1l1* mRNA expression in the testis by Northern blot analysis and fluorescence in situ hybridization (Fig. 1). In particular, *Got1l1* mRNA was localized in spermatocytes and sperms, but not in spermatogonia and Leydig cells. Got1l1 might be involved in the regulation of spermatogenesis. d-Aspartate was detected in elongate spermatids in the adult rat testis (Sakai et al. 1998). Thus, we examined the contents of some amino acids in the testis of WT and *Got1l1* KO mice by HPLC. However, the contents of d-aspartate and other examined amino acids were comparable between the two genotypes (Fig. 3), suggesting that Got1l1 is not a major d-aspartate-producing enzyme in the testis.

The Got1l1 protein is abundantly expressed in the brain and regulates neurogenesis in the adult hippocampus (Kim et al. 2010). In an immunohistochemical analysis, besides Got1l1-immunopositive signals, d-aspartate-immunopositive signals localized in the paraventricular nuclei, supraoptic nuclei, CA3/2 neurons of the hippocampus, and the hilus of the dentate gyrus (Kim et al. 2010). We attempted to produce an anti-Got1l1 antibody using recombinant Got1l1 expressed in *E. coli* as antigen; however, we were unable to obtain a specific antibody recognizing endogenous Got1l1. Some of the commercially available anti-Got1l1 antibodies could not be used because they detected same signal of bands in brain and testicular homogenate derived from WT and KO mice. Thus, we performed Northern blot analysis using total RNA from the brain, but were not able to detect *Got1l1* mRNA (Fig. 1). We were also unable to detect *Got1l1* mRNA signals in the adult hippocampus and other brain regions by fluorescence in situ hybridization (data not shown). Our analysis of the content of d-aspartate in the hippocampus of *Got1l1* KO mice showed no significant difference from that of WT mice. These findings suggest that expression level of *Got1l1* is low and Got1l1 is not a major d-aspartate-producing enzyme in the adult hippocampus. In contrast, the contents of l-glutamate and l-glutamine decreased in the adult hippocampus of *Got1l1* KO mice. The reasons underlying the reduced levels of l-glutamate and l-glutamine in the *Got1l1* KO hippocampus are currently unknown. One possibility is that *Got1l1* expressed in an early developing brain might be involved in amino acid metabolism.

The amino acid sequence of Got1l1 is closely related to those of Got family members, cytosolic Got1 and mitochondrial Got2 (Kim et al. 2010). Both Got1 and Got2 have conserved tryptophan at position 140, which prevents protonation of Cα for racemization of aspartate (Kim et al. 2010). Indeed, chicken-derived Got2 can generate d-aspartate; however, the speed of the reaction is very low; the rate of racemization of aspartate is 3.3 × 10^−8^ times the rate of transamination (Kochhar and Christen 1992). These findings suggest that members of the Got family are unlikely to be major aspartate racemases.

Recently, a novel dual racemase (DAR1) that can convert aspartate and serine to their chiral form in a PLP-dependent manner has been identified in *Aplysia* (Wang et al. 2011). DAR1 is 41 % identical to mammalian serine racemase; however, it is only 14 % identical to Got1l1, which did not show any aspartate racemase activity in our study. These findings suggest that the enzymatic pathways are conserved across the metazoan tree of life. One study demonstrated that the levels of d-aspartate in the forebrain are low in the serine racemase KO mice (Horio et al. 2013), which suggests the possibility of the involvement of serine racemase in d-aspartate production. Furthermore, in mammals, there are several poorly characterized serine dehydratase enzymes that show homology with serine racemase. We need to examine the activities of these enzymes and whether they produce d-aspartate in the steps to identify an unknown enzyme producing d-aspartate in mammals.

## Abbreviations

Got1l1: Glutamic-oxaloacetic transaminase-1 like 1
KO: Knockout
WT: Wild type
ES: Embryonic stem
SMG: Submandibular gland
PLP: Pyridoxal 5′-phosphate
*E. coli*: *Escherichia coli*
BAC: Bacterial artificial chromosome
GST: Glutathione *S*-transferase
